# Bile acid and cigarette smoke enhance the aggressive phenotype of esophageal adenocarcinoma cells by downregulation of the mitochondrial uncoupling protein-2

**DOI:** 10.18632/oncotarget.22380

**Published:** 2017-11-10

**Authors:** Yuan Xu, Paul L. Feingold, Deborah R. Surman, Kate Brown, Sichuan Xi, Jeremy L. Davis, Jonathan Hernandez, David S. Schrump, R. Taylor Ripley

**Affiliations:** ^1^ Thoracic and GI Oncology Branch, Center for Cancer Research, National Cancer Institute, Bethesda, Maryland 20852, USA

**Keywords:** bile acid, cigarette smoke, uncoupling protein-2, esophageal adenocarcinoma

## Abstract

Limited information is available regarding mechanisms that link the known carcinogenic risk factors of gastro-esophageal reflux and cigarette smoking to metabolic alterations in esophageal adenocarcinoma (EAC). In the present study, we utilized a novel *in-vitro* model to examine whether bile acid and cigarette smoke increase the aggressiveness of EAC and whether these changes are associated with metabolic changes.

EAC cells (EACC) were exposed to 10 μg/ml cigarette smoke condensate (CSC) and/or 100 μM of the oncogenic bile acid, deoxycholic acid (DCA), for 5 days. These exposure conditions were chosen given their lack of effect on proliferation or viability. DCA and CSC increased invasion, migration, and clonogenicity in EAC cells. These changes were associated with concomitant increases in ATP, ROS, and lactate production indicative of increased mitochondrial respiration as well as glycolytic activity. DCA and CSC exposure significantly decreased expression of uncoupling protein-2 (UCP2), a mitochondrial inner membrane protein implicated in regulation of the proton gradient. Knockdown of UCP2 in EACC phenocopied DCA and CSC exposure as evidenced by increased cell migration, invasion, and clonogenicity, whereas over-expression of UCP2 had an inverse effect. Furthermore, over-expression of UCP2 abrogated DCA and CSC-mediated increases in lactate and ATP production in EACC.

DCA and CSC promote the aggressive phenotype of EACC with concomitant metabolic changes occurring via downregulation of UCP2. These results indicate that UCP2 is integral to the aggressive phenotype of EACC. This mechanism suggests that targeting alterations in cellular energetics may be a novel strategy for EAC therapy.

## INTRODUCTION

Esophageal adenocarcinoma (EAC) is the dominant esophageal cancer histology in the United States with an average annual increase of 6% [1]. Despite some advances, the 5-year survival rate for all patients diagnosed with esophageal cancer remains less than 20%. There is an urgent need to develop effective and novel approaches for treatment.

Environmental risk factors play a significant role in the development and progression of EAC. Gastro-esophageal reflux disease and cigarette smoking are major risk factors for esophageal adenocarcinoma [2, 3]. Along with gastric acid, bile acids enter the esophagus during episodes of reflux [4, 5]. The most common bile acids are cholic acid, deoxycholic acid (DCA), chenodeoxycholic acid (CDCA), glycocholic acid, taurocholic acid, lithocholic acid, and ursodeoxycholic acid. DCA and CDCA are the main functional components in gastric reflux [6]. DCA is a secondary bile acid that is the most closely linked to EAC. DCA is cytotoxic to esophageal cells and may contribute to esophageal carcinogenesis [7]. DCA induces abnormal expression of genes relevant to cell survival, proliferation, invasion, and metastasis [8]. Cigarette smoking is another leading risk factor [2, 5, 9]. Although the association of cigarette smoke with the development of EAC is much less robust, cigarette smoking is still a known risk factor and has been implicated in the prognosis of patients with EAC [10–12]. Little is known about the pathogenic mechanisms by which bile acid or cigarette smoke induce EAC progression. Therefore, we developed an *in-vitro* model to study these mechanisms.

Altered energy metabolism is a ubiquitous phenomenon in cancer cells and an accepted hallmark of cancer [13]. Many cancer cells exhibit a metabolic shift from ATP generation through oxidative phosphorylation (OXPHOS) to ATP generation through aerobic glycolysis even under normal oxygen concentrations (Warburg effect) [14]. Aerobic glycolysis is associated with high lactate production which has been implicated in invasiveness and metastasis. The decrease in ATP production at first appears counterintuitive, but the metabolic alterations facilitate biosynthesis of macromolecules necessary to meet the sustained growth of cancer cells [15]. The mitochondrion is the critical organelle involved in metabolism and bioenergetics and plays an essential role in the redirection of substrates for macromolecule synthesis [16]. Additionally, the mitochondrion is the main source of ROS production. Excessive ROS can initiate cellular damage associated with increase cancer progression [17]. Thus, an understanding of metabolic changes in EAC may elucidate pathways associated with progression.

UCP2 is a member of the uncoupling protein family, which belongs to the mitochondrial anion transporter superfamily located in the inner mitochondrial membrane [18]. ATP generation occurs by the electron transport chain (ETC) protein, ATP synthase (also called Complex V). ATP synthase requires a proton gradient across the inner mitochondrial membrane from the inner mitochondrial membrane space to the matrix. UCP2 uncouples OXPHOS via dissipation of the proton gradient across the mitochondrial inner membrane to bypass ATP Synthase [19]. UCP2 decreases the efficiency of mitochondrial ATP production and thereby limiting ROS generation which provides protection against excessive ROS stress. UCP2 has been reported to have functions other than as an uncoupling protein. Several studies have reported that UCP2 acts as metabolic regulator by altering glucose metabolism [20–22]. Some reports have indicated that UCP2 acts as a tumor suppressor because it has been associated with decreased proliferation and malignant progression in cancer cells [22–24]. Collectively, these findings suggest that the functions of UCP2 may be contingent on tissue specificity.

To evaluate altered cellular energetics in esophageal cancer, the current study used an *in-vitro* model to examine the effects of DCA and CSC on EAC cells. We surmised that if DCA and CSC altered the malignant phenotype of EACC, that a concurrent change in the cellular energetics would occur. We hypothesized that targeting aberrant cellular energetics may be a novel strategy for the treatment of esophageal carcinoma.

## RESULTS

### Bile acid and cigarette smoke enhance the aggressive phenotype of EACC

**Figure 1:**
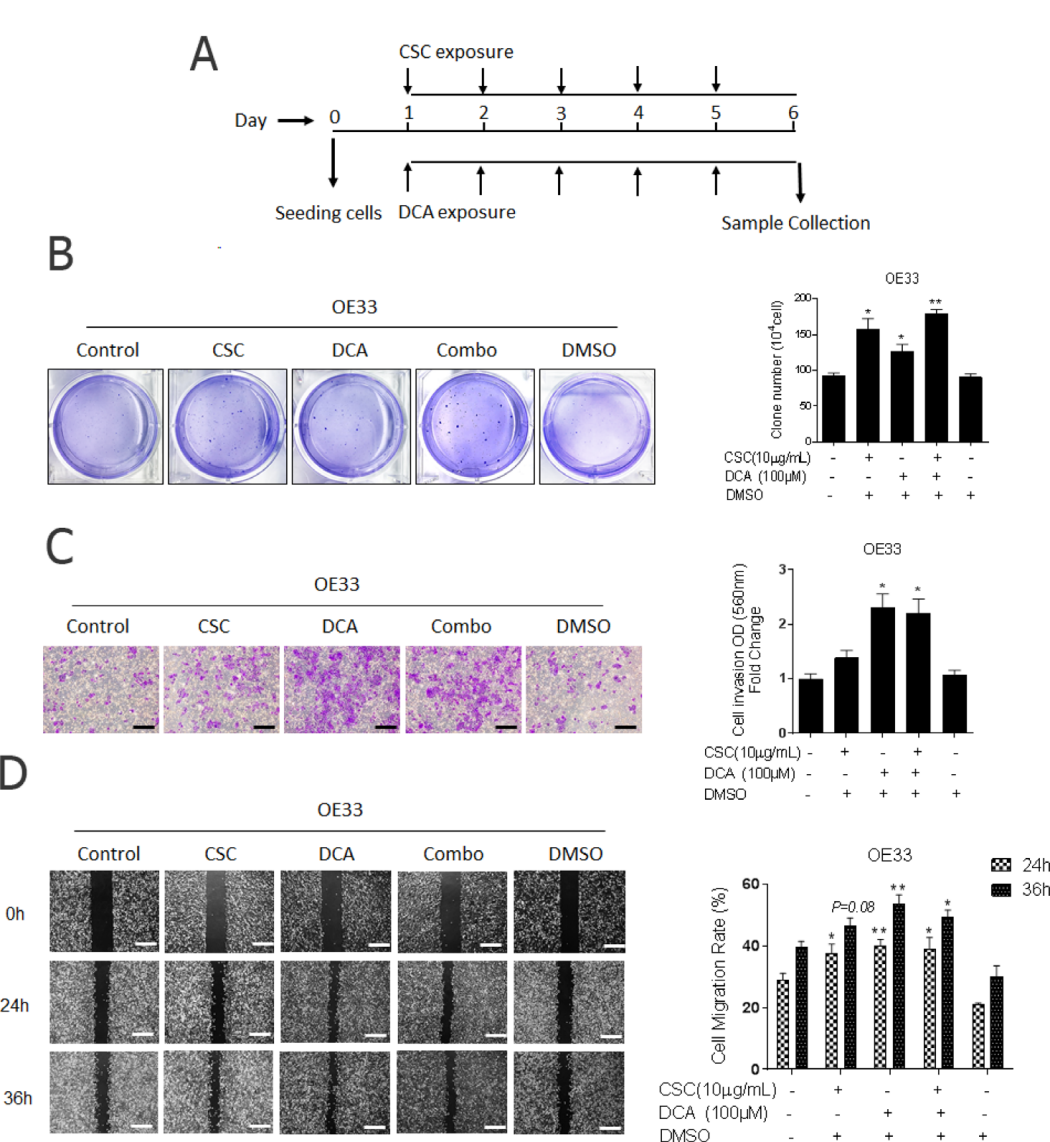
Bile acid and cigarette smoke enhance the aggressive phenotype of esophageal adenocarcinoma cells (**A**) Cigarette smoke and bile acids exposure model: Cells were cultured in 10-cm dishes in appropriate normal media (NM), NM with DMSO, NM with CSC (10 μg/ml), NM with DCA (100 μM), or NM with combination of DCA and CSC for 5 days. Medium was changed daily with the addition of fresh CSC, DCA, or DMSO control. Cells were harvested at the 6th day for further analysis. Esophageal adenocarcinoma cell line, OE33 cells were cultured in these conditions. (**B**) The colonies in soft agar were stained with crystal violet, photographed, and counted to quantify (mean ± SD). Three independent experiments were performed. (**C**) Invasion was determined by transwell assays. Representative image of invading cells is shown (original magnification × 100, scale bar = 50 μm). Quantitative analysis of invasion was measured by absorbance at OD 560 nm after staining of invading cells with crystal violet. Fold changes (mean ± SD) were obtained from three independent experiments. (**D**) The cell migration was analyzed wound-healing assays in OE33 cells. Photographs were obtained at 0 h (immediately after scratching) and at the indicated time intervals shown (original magnification × 40, scale bar = 125 μm). Covered areas by migrated cells in the nine random fields after exposure for 0, 24, 36 h were quantified by Image J software. ^*^P < 0.05 and ^**^P < 0.01 as compare with non-treatment group.

EACC (OE33, FLO-1, and Esc2) cells were exposed to 10 μg/ml CSC and/or 100 μM DCA for 5 days. We changed the condition medium each day (Figure 1A). Given that unregulated cell proliferation leads to malignant progression and invasive behavior [13], these concentrations alone and in combination were chosen due to their negligible effects on proliferation and viability of these cells in either RPMI and DMSO (Supplementary Figure 1). Similar growth rates suggest that CSC and DCA induction of EAC malignant progression was dissociated from cell proliferation. To investigate the effects of DCA and CSC on the aggressive phenotype of the cells, anchorage-independent growth (clonogenicity), cell invasion, and migration were evaluated in EACC. In the clonogenicity assays, the number and size of colonies increased by 1.5- fold in CSC single treated cells, while the effects of DCA were less robust but still statistically significant (Figure 1B). The combination of DCA and CSC treated cells showed the highest clonogenicity. After 24 h incubation in the transwell invasion assay, the DCA or combination treated cells had a significant increase in invasion (Figure 1C). Cells treated with DCA alone, CSC alone, or the combination all showed increased migration at both 24 and 36 hours compared to controls (Figure 1D). All these aggressive phenotypic changes were also found in other EACC lines (Supplementary Figure 2). The findings of increased invasion, migration, and clonogenicity suggest that DCA and CSC, both independently and in combination, increase the aggressive phenotype of EACC.

### Bile acid and cigarette smoke alter mitochondrial function and promote glycolysis

Dysregulation of cellular metabolism has been associated with malignant transformation [25]. The shift in the metabolism may be reflected in cellular ATP levels and lactate production [26]. Given the increase in aggressive phenotype in DCA and CSC treated cells, we asked whether these aggressive phenotypic changes were associated with altered mitochondrial function. Although cancer cells are characterized in general by a decrease in OXPHOS with a strong enhancement of glycolysis (Warburg effect) [14], there are numerous publications in which cancer cells have been reported to show normal or even high OXPHOS [16, 27].

**Figure 2:**
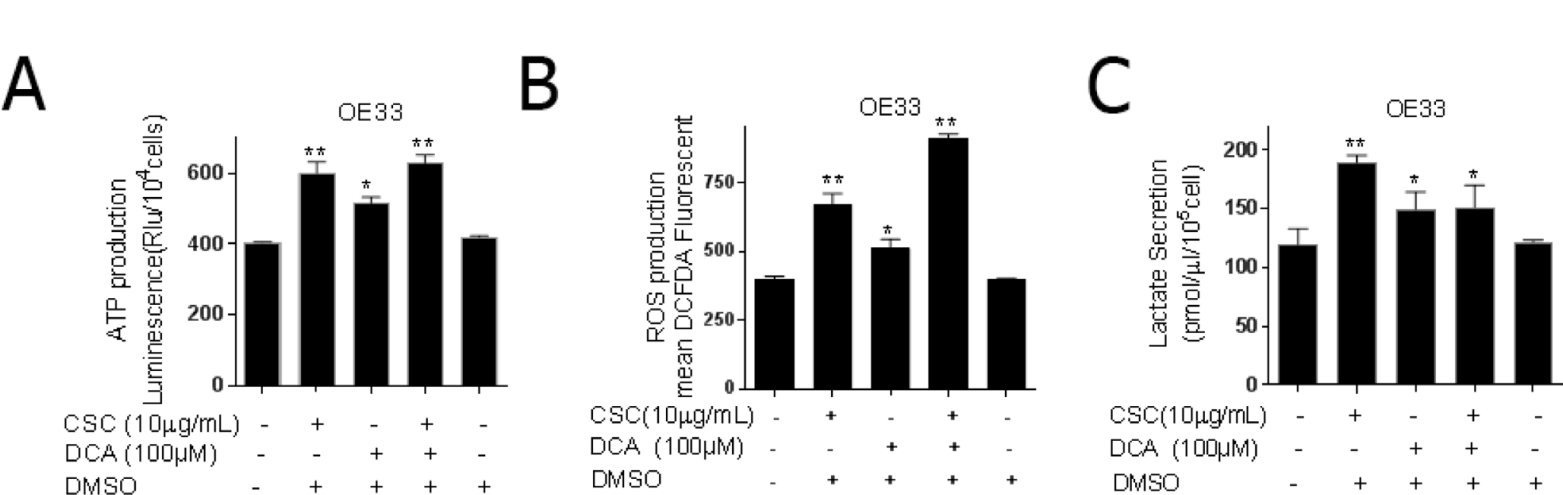
Bile acid and cigarette smoke alter mitochondrial function and promote glycolysis OE33 cells were cultured in the presence or absence of CSC and/or DCA for 5 days. (**A**) ATP levels were measured by luciferase luminescence intensity which was normalized to the cell number (mean ± SD) from three independent experiments. (**B**) The level of cellular ROS concentrations was measured using DCFDA fluorescence by flow cytometry with the geometric mean of fluorescence ± SD analyzed using Cell Quest software. (**C**) Lactate released into the culture medium were measured by absorbance at OD 560 nm and normalized to the cell numbers (mean ± SD) from three independent experiments. ^*^P < 0.05, and ^**^P < 0.01 as compare with control culture group.

Indeed, CSC and DCA alone and in combination increased ATP production in OE33 cells which is indicative of increased OXPHOS (Figure 2A) [28]. We then asked whether these changes were associated with an increase in ROS. We observed that CSC and DCA treatment independently and in combination increased ROS production in OE33 cells (Figure 2B). Similar findings were noted in Flo-1 cells (Supplementary Figure 3). These findings suggest that as opposed to downregulation of OXPHOS noted in the Warburg effect, that mitochondrial function is enhanced. Complimentary to OXPHOS, we queried whether changes in glycolysis occurred with CSC and DCA. CSC and DCA treated OE33 cells had significantly higher lactate secretion compared to non-treated cells (Figure 2C). Similar findings were noted in Flo-1 cells (Supplementary Figure 3). Based on the enhancement of ATP production, ROS generation, and lactate secretion, DCA and CSC exposure altered metabolic function of EACC.

### Bile acid and cigarette smoke condensate decreased UCP2 expression

To further evaluate the molecular mechanisms governing CSC and DCA-induced metabolic changes, we screened multiple metabolically-associated genes of interest by qRT-PCR and immunoblots (Supplementary Figure 4). We observed significant changes in expression levels of mitochondrial inner membrane protein, uncoupling protein 2 (UCP2). We also observed changes in the first and rate-limiting protein of glycolysis, hexokinase 2 (HK2) [19, 29]. UCP2 was specifically tested because its expression is inversely related to ROS generation, therefore, reduction in this protein could account for the increased in ROS observed with CSC and DCA.

**Figure 3:**
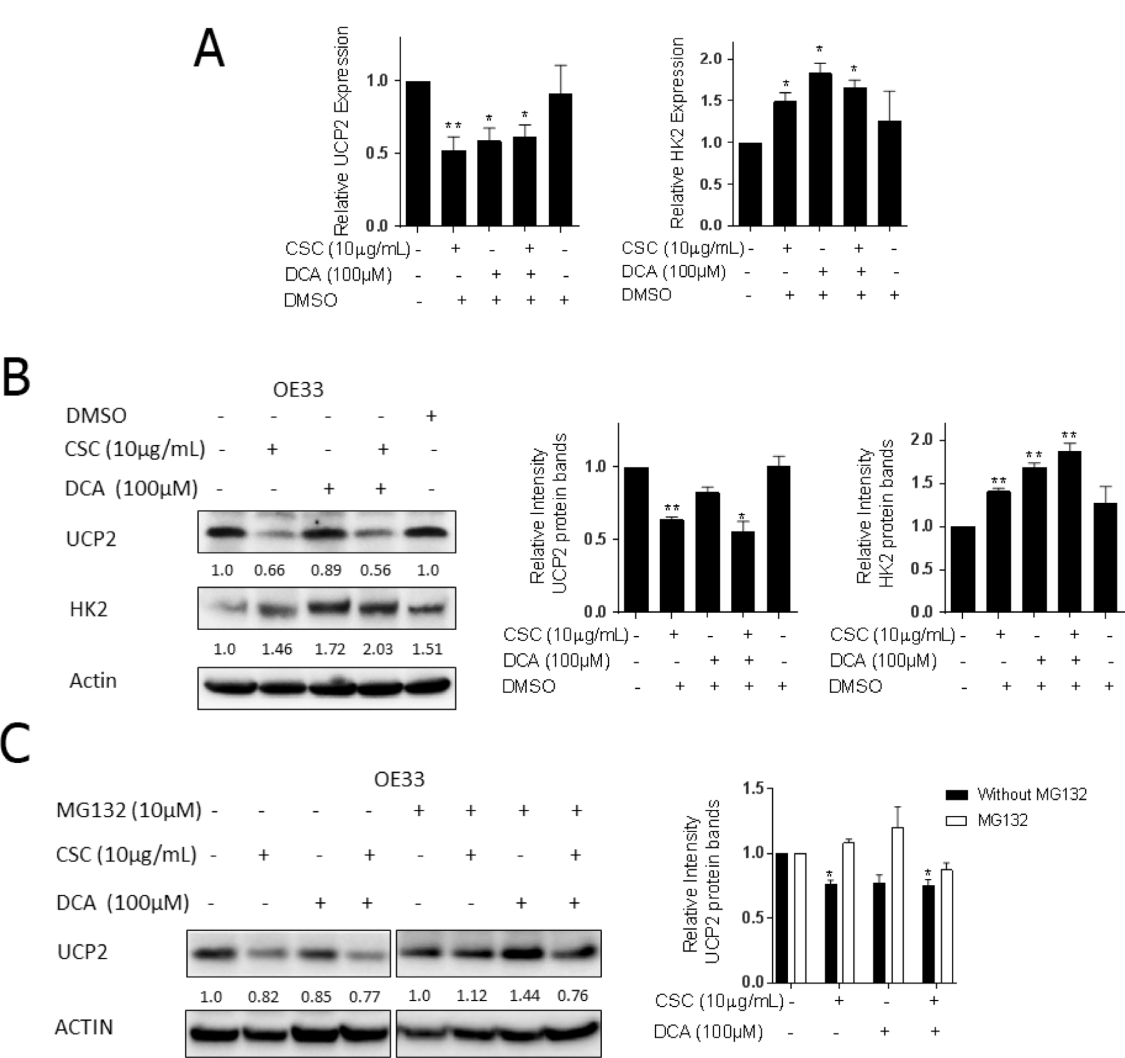
Bile acid and cigarette smoke condensate inhibit UCP2 expression and increase HK2 expression Repression of UCP2 is secondary to protein degradation. OE33 cells cultured in the presence or absence of CSC and/or DCA for 5 days. (**A**) qRT-PCR analysis of UCP2 and HK2 expression normalized with actin in OE33 cells. (**B**) The protein levels of UCP2 and HK2 were analyzed by Western blots. The relative protein levels of UCP2 were quantified by Image lab software and corrected for loading control β-actin. Quantification of UCP2 and HK2 expression is expressed relative to control group. (**C**) OE33 cells were treated with 10.0 μM proteasome inhibitor MG132 for 6 h after 5 days of exposed to CSC and DCA. Levels of UCP2 were determined by western blots in the absence or presence of MG132. The relative protein levels of UCP2 were quantified by Image lab software and corrected for loading control β-actin. Quantification of UCP2 expression is expressed relative to control group. ^*^P < 0.05, and ^**^P < 0.01 as compare with control culture group.

We observed that both protein and mRNA levels of UCP2 were decreased in OE33 cells while HK2 protein levels were increased (Figure 3A and 3B). The same patterns were found in other EACC lines (Supplementary Figure 5A). To further verify the specificity of UCP2 antibody, we applied UCP2 WT and KO mice to test UCP2 expression with UCP2 antibody, the results showed that UCP2 expressed in WT mice, but no any signal observed in UCP2 KO mice (Supplementary Figure 4D). There are various molecular mechanisms that have been reported to regulate UCP2, including transcriptional, translational, and posttranslational modifications [30]. We noted that the UCP2 protein depletion was more pronounced than the decrease in mRNA levels, therefore, we explored translational and posttranslational regulation of UCP2. We screened miRNAs targeted to the promoter of UCP2, then tested potential miRNAs expression in our exposure model. We did not observe any effects of CSC and DCA on miRNA expression (data not shown).

We next explored the possibility that a UCP2 depletion in EACC following CSC and DCA exposure was mediated by proteolytic degradation [31]. Notably, the proteasome inhibitor, MG132, rescued UCP2 repression induced by CSC and DCA (Figure 3C). To further examine if the reduction of UCP2 protein levels in CSC and DCA treated EACC was due to protein degradation, we exposed these cells to cycloheximide (CHX, 10 μg/ml), a protein synthesis inhibitor. Briefly, EACC were pretreated with CSC and DCA for 5 days followed by the addition of CHX for 0.5–4 hours. The rate of UCP2 disappearance was determined. In non-treated control cells, the UCP2 levels were still stable from 1 to 4 hours. In contrast, in the CSC and DCA treated cells, UCP2 had a shorter half-life and its degradation was augmented. The protein was reduced by almost 50% 4 hours after CHX treatment (Supplementary Figure 5). These results indicate that CSC and DCA induce UCP2 depletion by promoting its degradation.

### UCP2 impairs aggressive phenotype of EACC

**Figure 4:**
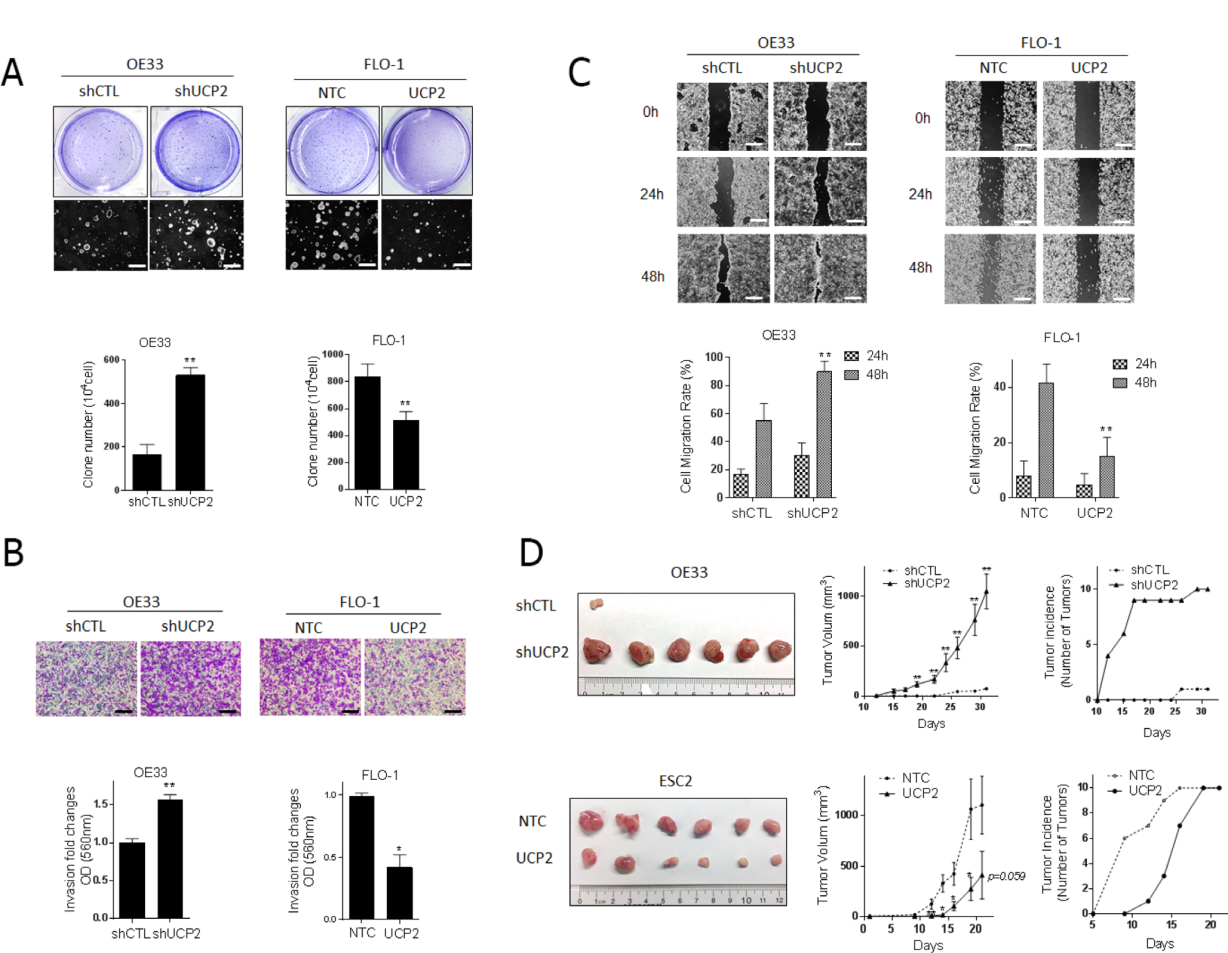
UCP2 impairs malignant progression of esophageal cancer cells Phenotypic assays were performed in FLO-1 and Esc2 cells stably expressing non-targeting control (NTC). OE33 cells were generated with stable knock-down of UCP2 (shUCP2) or scrambled control (shCTL) by transfecting short-hairpin RNA lentiviruses. (**A**) The colonies in soft agar were stained with crystal violet, photographed (original magnification × 20, scale bar = 250 μm), and counted to quantify (mean ± SD). Three independent experiments were performed. (**B**) Invasion was determined by transwell assays. Representative image of invading cells is shown (original magnification × 100, scale bar = 50 μm). Quantitative analysis of invasion was measured by absorbance at OD 560 nm after staining of invading cells with crystal violet. Fold changes (mean ± SD) were obtained from three independent experiments. (**C**) The cell migration was analyzed with wound-healing assays. Photographs were obtained at 0 h (immediately after scratching) and at the indicated time intervals shown (original magnification × 40, scale bar = 125 μm). Covered areas by migrated cells in the nine random fields after exposure for 0, 24, 48 h were quantified by Image J software. ^*^P < 0.05, ^**^P < 0.01 as compare with indicated control group. (**D**) Xenograft experiments were performed in OE33 cells with stable knock down of UCP2 (shUCP2) or scrambled control (shCTL), and Esc2 cells with UCP2 or control transfection, cells were injected subcutaneously into flanks of nude mice (10 mice/20 flanks per experiment). Photographs of representatives harvested tumors derived from each group. Tumor growth curve in nude mice measured by caliper (mean ± SD), and tumor incidence was calculated. Two independent experiments were performed and gave similar results. ^*^P < 0.05, and ^**^P < 0.01 as compare with control group.

Given that DCA and CSC altered the expression of UCP2, we queried whether blocking UCP2 recapitulated the phenotypic effects associated with DCA and CSC. Additionally, we asked whether overexpression of UCP2 could reduce the aggressive phenotype of these cells. Cell proliferation, clonogenicity, wound healing, transwell invasion assays, and xenograft tumorigenicity assays were performed. UCP2 was either knocked down or overexpressed in EACC (Supplementary Figure 6). Neither UCP2 overexpression nor downregulation induced any effects on proliferation of EACC relative to control cells (Supplementary Figure 7). The lack of proliferative effects is important because it suggests that changes in other assays are not secondary to an increase in total number of cells, but instead are secondary to other phenotypic alterations. In colony-forming assays, UCP2 knockdown resulted in a >2-fold increase in clone number. In contrast, UCP2 overexpression significantly decreased clonogenicity compared to controls (Figure 4A). Similarly, knockdown of UCP2 significantly increased whereas UCP2 overexpression significantly decreased invasion, respectively (Figure 4B and Supplementary Figure 8A). Wound healing assays revealed that the knockdown of UCP2 accelerated wound closure, whereas UCP2 overexpression, virtually abolished migration of EACC over 48 hours (Figure 4C and Supplementary Figure 8B).

Additional experiments were performed to examine the effects of UCP2 expression on tumorigenesis *in-vivo*. Only one of 10 nude mice inoculated subcutaneously with control OE33 cells developed a tumor, which was quite small. In contrast, 10 of 10 mice inoculated with UCP2 knockdown OE33 cells developed tumors which were uniformly much larger than the one control tumor (Figure 4D). Similar results were also found in UCP2 knockdown FLO-1 cell (Supplementary Figure 8C). Because FLO-1 cells exhibited lower tumorigenicity compared with Esc2 cells, we chose Esc2 cells to test tumor volumes and incidence following UCP2 overexpression. As shown in Figure 4D lower panel, tumor volumes of subcutaneous xenografts established from Esc2 cells constitutively expressing UCP2 were significantly smaller than tumors derived from vector control cells. In Esc2 cells, although all mice had tumors at the end of experiment, most control xenografts whereas detectable within 2 weeks while UCP2 overexpressing cells exhibited delayed tumor formation. Collectively, these findings suggest that UCP2 acts as a tumor suppressor and is tightly linked to the aggressive phenotype of EACC.

### UCP2 modulates mitochondrial function and glycolysis in EACC

**Figure 5:**
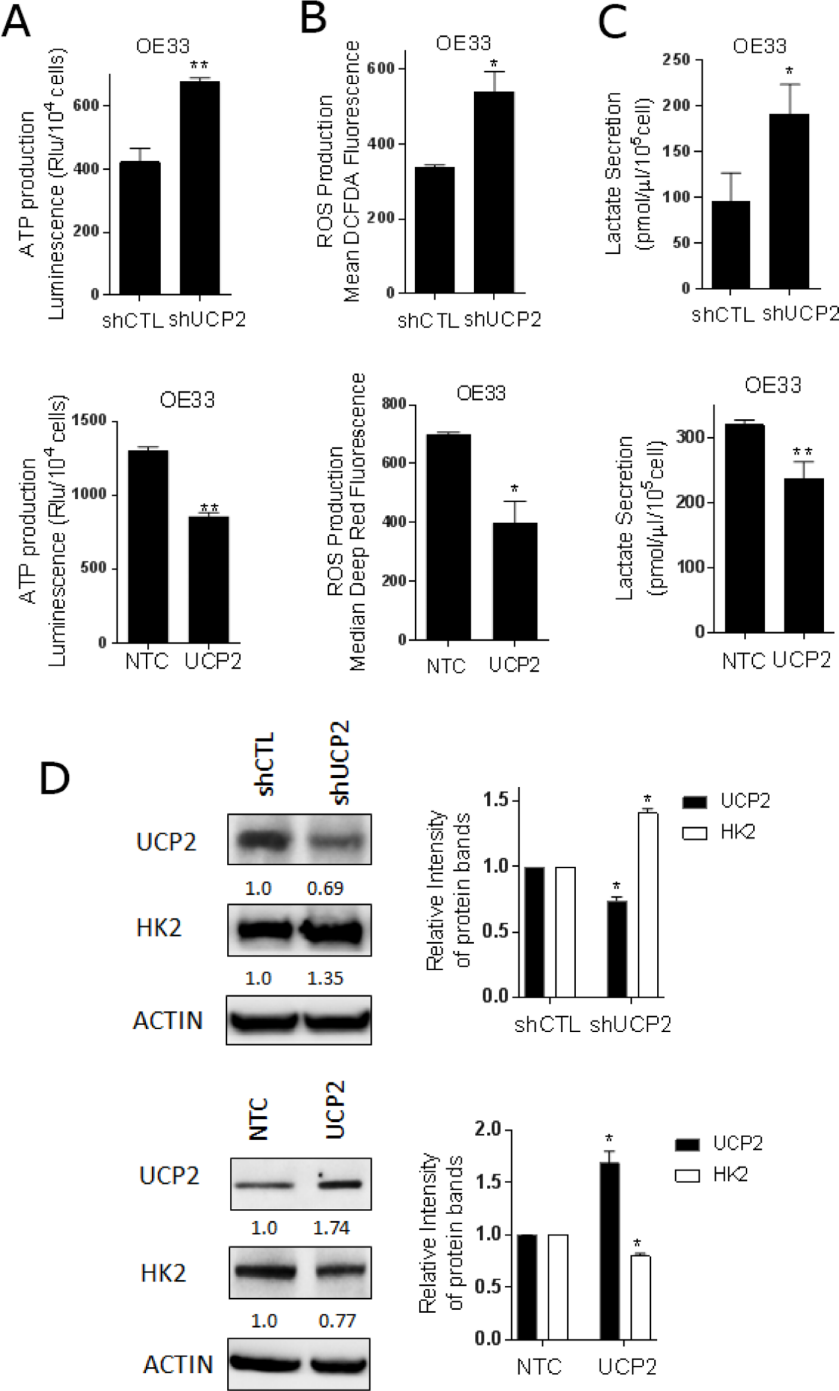
UCP2 modulates mitochondrial function and glycolysis in esophageal cancer cells Mitochondrial and glycolytic assays were performed in OE33 cells with stable knock down of UCP2 (shUCP2) or scrambled control (shCTL) by transfecting short-hairpin RNA lentiviruses. OE33 cells were stably generated expressing non-targeting control (NTC) or UCP2 by transfecting lentiviruses. (**A**) ATP levels were measured by luciferase luminescence intensity which was normalized to the cell number (mean ± SD) from three independent experiments. (**B**) The level of cellular ROS concentrations was measured using DCFDA fluorescence and Deep Red fluorescence by flow cytometry with the geometric mean of fluorescence ± SD analyzed using Cell Quest software. (**C**) Lactate released into the culture medium were measured by absorbance at OD 560 nm and normalized to the cell number and to protein concentration (mean ± SD) from three independent experiments. (**D**) Western analysis of UCP2 and HK2 expression in UCP2 knockdown and overexpressed cells. The relative protein levels of UCP2 were quantified by Image lab software and corrected for loading control β-actin. Quantification of UCP2 and HK2 expression is expressed relative to indicated control group. ^*^P < 0.05, and ^**^P < 0.01 as compare with indicated control group.

Given that UCP2 is an inner mitochondrial membrane protein that decreases the proton gradient across this membrane, we queried whether the expected changes occurred with differential expression of UCP2. To review, decreases in UCP2 will increase the proton gradient between the inner membrane space and the matrix, which will enable ATP synthase (complex V of the ETC) to produce more ATP. As a byproduct of the ETC, ROS levels increase [32]. UCP2 is reported to serve as a ‘pop-off valve’ that limits the proton gradient and protects the cells by reducing the ROS generation. Indeed, these expected changes were noted in EACC. With knockdown of UCP2, ATP levels were increased, whereas in UCP2 overexpressing cells, ATP levels were decreased (Figure 5A). Consistent with these findings, we observed higher ROS production with lower UCP2 expression and lower ROS production with higher UCP2 expression compared to the controls (Figure 5B). These results indicate that UCP2 alters mitochondria function in EAC cells as reported in the literature [19, 33].

To extend these findings beyond the expected mitochondrial functions, lactate production was evaluated. Interestingly, lactate accumulation was significantly higher in UCP2 knockdown cells compared with control cells, whereas, lactate was lower in EACC overexpressing UCP2 (Figure 5C). Therefore, we analyzed the expression of glycolytic enzymes in cells transfected with UCP2 lentivirus. The results revealed that downregulation UCP2 modestly increased HK2 protein expression with no obvious effects on other tested enzymes (Figure 5D). The increase in HK2 expression with knockdown of UCP2 was similar to the increases of HK2 with DCA and CSC treatment. These findings suggest that the function of UCP2 are not restricted to the mitochondria, but also influence the glycolytic pathway.

### UCP2 is integral to bile acid and cigarette smoke induced metabolic changes of EACC

**Figure 6:**
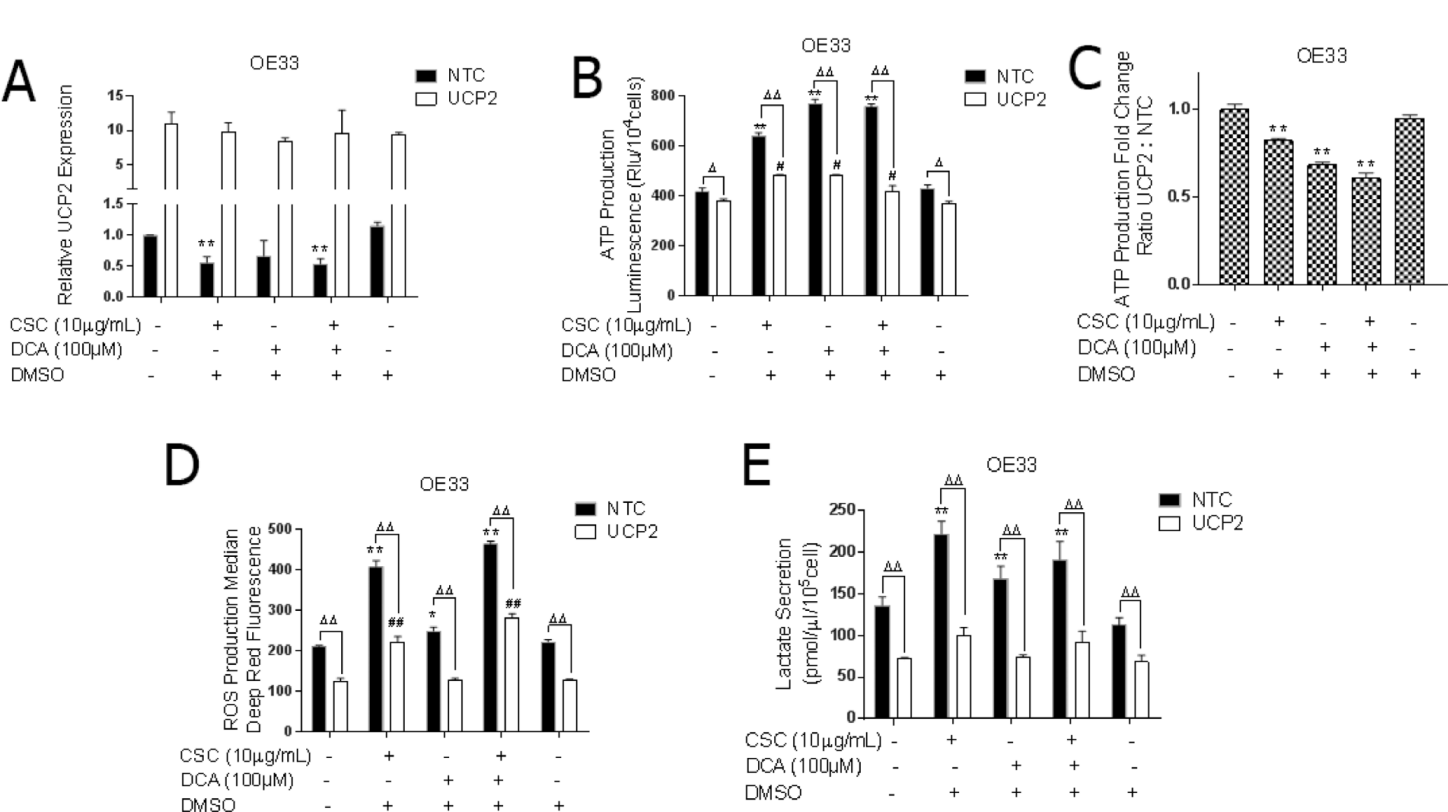
UCP2 is integral to bile acid and cigarette smoke induced metabolic reprogramming of EAC cells OE33 cells stably expressing non-targeting control (NTC) or UCP2 were cultured in the presence or absence of CSC and/or DCA for 5 days. (**A**) qRT-PCR analysis of UCP2 expression normalized with actin. ^*^P < 0.05, and ^**^P < 0.01 as compare with non-treated NTC cells. (**B**) ATP levels were measured by luciferase luminescence intensity which was normalized to the cell number (mean ± SD). ^*^P < 0.05, and ^**^P < 0.01 as compare with non-treated NTC cells. ^#^*P* < 0.05, and ^##^*P* < 0.01 as compare with non-treated UCP2 overexpressed cells. ^Δ^*P* < 0.05, and ^ΔΔ^*P* < 0.01 as compare with NTC cells. (**C**) Ratio of ATP production comparing the increased fold change in UCP2 overexpressed cells to the increased fold change in NTC cells. ^*^P < 0.05 as compare with control group. (**D**) The levels of cellular ROS concentrations were measured using DCFDA fluorescence by flow cytometry with the geometric mean of fluorescence ± SD analyzed using Cell Quest software. ^*^P < 0.05, and ^**^P < 0.01 as compare with non-treated NTC cells. ^#^*P* < 0.05, and ^##^*P* < 0.01 as compare with non-treated UCP2 overexpressed cells. ^Δ^*P* < 0.05, and ^ΔΔ^*P* < 0.01 as compare with NTC cells. (**E**) Lactate released into the culture medium were measured by absorbance at OD 560 nm and normalized to the cell number and protein concertation (mean ± SD) from three independent experiments in UCP2 overexpressed OE33 cells after CSC and DCA exposure for 5 days. ^*^P < 0.05, and ^**^P < 0.01 as compare with non-treated NTC cells. ^Δ^*P* < 0.05, and ^ΔΔ^*P* < 0.01 as compare with NTC cells.

To assess whether UCP2 overexpression could abrogate the metabolic changes induced by DCA and CSC exposed cells, OE33 cells overexpressing UCP2 were exposed to DCA and CSC for 5 days. We first checked whether UCP2 expression was altered in the UCP2 overexpressing cells upon DCA and CSC exposure, and observed no downregulation of UCP2 (Figure 6A). As noted earlier, ATP production increased with DCA and CSC exposure and decreased with UCP2 overexpression. When OE33 UCP2 expressing cells were exposed to DCA and CSC, the production of ATP was significantly abrogated compared to controls. ATP production was increased by nearly 2-fold in the control cells, but only 1.2-fold in UCP2 overexpressors (Figure 6B and 6C). These findings suggested that UCP2 can almost completely block CSC- and DCA-induced ATP production. Similarly, CSC and DCA exposure only modestly changed the ROS levels in EACC overexpressing UCP2, whereas ROS levels increased by 2-fold in control cells (Figure 6D).

Next, we examined if over-expression of UCP2 could modulate lactate production in EACC exposed to DCA and/or CSC. Interestingly, the increase in lactate secretion induced by CSC and DCA was completely blocked by UCP2 overexpression (Figure 6E). Collectively, these results demonstrate that downregulation of UCP2 results in metabolic reprogramming which increases mitochondrial respiration as well as glycolysis in EACC.

## DISCUSSION

We established an *in-vitro* model of the well-known esophageal adenocarcinoma risk factors, bile acid and cigarette smoke, to query whether they increased the aggressive phenotype of EACC and, if true, whether this cellular phenotype was associated with metabolic changes. We observed that both DCA and CSC increased clonogenicity, invasion, and migration in EACC without increases in proliferation. Next, we observed changes in metabolism based on increases in ATP production, ROS generation, and lactate secretion. We tested the expression of several well-known enzymes involved in metabolism and noted a significant reduction in UCP2. We particularly focused on UCP2 because of its reported mitochondrial function could account for the alterations in ROS generation. We explored the function of UCP2 in EACC and noted that the expected mitochondrial changes to ATP production and ROS generation occurred. More interestingly, UCP2 acted as a tumor suppressor and altered glycolysis in addition to the mitochondrial effects in EACC. Furthermore, UCP2 nearly completely abrogated the effects associated with DCA and CSC. Taken together, these findings strongly suggest that DCA and CSC enhance progression of EAC via perturbations of energy production. Collectively these findings provide rationale for exploring novel therapeutics based on targeting metabolic alterations in these neoplasms.

Although only relatively recently defined as a hallmark of malignancy, metabolic alterations in cancer cells were described by Dr. Warburg nearly a century ago. He reported that cancer cells prefer to produce ATP by glycolysis, which is a less efficient pathway compared to OXPHOS, a phenomenon known as the Warburg effect [14]. We established an *in-vitro* model to specifically evaluate metabolic changes associated with EACC. Once we established that the model could induce a more aggressive phenotype in EACC, we tested whether these cells underwent a Warburg effect from energy production in the mitochondria by OXPHOS toward glycolysis. We observed increases in lactate secretion suggesting that glycolysis was upregulated, but we also observed increases in ATP production and ROS generation suggesting an increase in OXPHOS. The increases in glycolysis are consistent with a Warburg effect, but the increases in mitochondrial respiration are not. Although the decrease of OXPHOS is reported by many researchers as a universal feature of malignant cells, other studies have revealed the existence of a diverse class of malignant cancer cells in which ATP is produced at a higher level by mitochondrial OXPHOS [34]. The work by Moreno-Sanchez *et al.* showed that mitochondrial impairment does not seem to apply in nonhypoxic, oxidative tumors [27, 34, 35]. They suggest that glycolysis is not driven to maintain energy demands to overcome defective mitochondria; rather, other mitochondrial alterations such as substrate oxidation, ROS production, or mitochondrial DNA mutations may reprogram the mitochondria to assist in malignant progression. Additionally, the mitochondria have multiple functions not associated with the generation of ATP, which include production of the macromolecules such as proteins, nucleotides, and lipids that are necessary for continuous tumor cell division. Our results indicate that DCA and CSC induce metabolic changes without impairment of mitochondrial respiration or OXPHOS. These findings support the notion that the mitochondria are not dysfunctional, but rather are functionally reprogrammed to support the needs of a progressive cancer cell.

We observed that DCA and CSC increased ROS generation. Given that overproduction of ROS can initiate damage to macromolecules and to intracellular and extracellular signaling pathway associated with survival and malignancy properties, we explored known mitochondrial mechanisms that may account for changes in ROS [17]. As noted above, UCP2 expression was significantly reduced by DCA and CSC, which could account for the changes in ROS [19]. Based on both overexpression and knockdown of UCP2, our results indicate that UCP2 alters ATP production and ROS as expected based on its uncoupling activity (Figure 5A and 5B) [36]. UCP2's role in uncoupling activity has been debated. Evidence supporting an uncoupling function for UCP2 include its homology to UCP1 and increased respiration in yeast expressing the protein [19]. In beta cells, the absence of UCP2 abolished alternative pathways of proton return in the matrix, increased mitochondrial membrane potential, increased ATP levels, closed KATP channels, and stimulated insulin secretion [37]. The absence of an uncoupling protein should result in an increase the mitochondrial membrane potential with a concomitant increase in ROS production. In contrast to these expected findings, some studies reported that proton conductance occurs in normal hepactocytes in the absence of UCP2 or other uncoupling proteins under physiological conditions [38]. In the present study, we believed that UCP2 functions as an uncoupling protein and its expression is altered by CSC and DCA. Additionally, we found that CSC and DCA did not change almost mitochondrial ETC expression which supports that ATP generation is increased secondary to UCP2 inhibition (Supplementary Figure 9A). Moreover, knockdown of UCP2 increased the mitochondrial membrane potential (^Δ^ψm) which reinforces the conclusion that UCP2 alters ATP production and ROS as expected based on its uncoupling activity (Supplementary Figure 9B).

Besides the uncoupling protein in mitochondrial function regulation, UCP2 may have additional metabolic effects. UCP2 is thought to be involved in the coupling between glucose oxidation and mitochondrial biogenesis by the promotion of mitochondrial fatty acid oxidation. In UCP2 -/- cells, fatty acid entry into mitochondria is decreased, thereby increasing the utilization of glycolytic-derived pyruvate to fuel mitochondria [24]. Parton *et al.* have shown that UCP2 negatively regulates glucose sensing in neurons and its absence prevents obesity-induced loss of glucose sensing [39]. Overexpression of UCP2 has been reported to trigger metabolic reprogramming favoring oxidative metabolism with increased expression of pyruvate dehydrogenase and OXPHOS and decreased expression of HK2 and pyruvate kinase isoform 2 enzymes [22]. Our experiments demonstrate that expression of UCP2 negatively regulated glycolysis as noted by decrease in lactate production. Consistent with these findings, knockdown of UCP2 significantly increased HK2 protein expression, whereas, overexpression reduced HK2 expression. HK2 is the rate-limiting step of glycolysis and its expression can account for the changes in lactate production. Although we could not elucidate the exact mechanism by which UCP2 affected HK2 expression, our results are consistent with others showing that UCP2 functions as a glycolytic regulator in addition to the uncoupling role in the mitochondria. UCP2 overexpression completely blocked CSC and DCA induced increases in lactate secretion. These results suggest that the mitochondria have an influential role in glycolytic regulation through UCP2 and the mitochondria and UCP2 as important mediators of aggressive phenotype of EACC.

The precise biologic functions of UCP2 in cancer regulation remain subject of debate. UCP2 over-expression has been found in some cancers, including bladder, colorectal, kidney, and breast cancer [20, 21]. In contrast, other reports suggest that UCP2 is a tumor suppressor and its re-expression is a potential therapeutic strategy [22, 24]. For example, UCP2-null mice are more susceptible to carcinogens as noted by enhanced tumorigenesis in the proximal colon [40]. Additionally, the repression of UCPs by estrogens, a major risk factor for breast cancer initiation, plays a key role in estrogen-induced breast carcinogenesis [41]. Our observations support UCP2's role as a tumor suppressor. We noted a significant reduction in clonogenicity, invasion, migration, and tumor formation and size with overexpression of UCP2. Conversely, knockdown of UCP2 had the opposite effects. The expression of UCP2 could not be unlinked from ROS generation, therefore, the alterations in the aggressive phenotype of EACC may not be independent of ROS. Additionally, we explored the mechanism of UCP2 downregulation and observed that the proteasome inhibitor, MG132, could prevent the degradation of UCP2. UCP2 has a short half-life and has been reported to be degraded by the cytoplasmic ubiquitin-proteasome despite its location in the inner mitochondrial membrane [30, 42, 43]. Therefore, we believe that rapid proteasome degradation is responsible for the UCP2 decrease induced by DCA and CSC.

**Figure 7:**
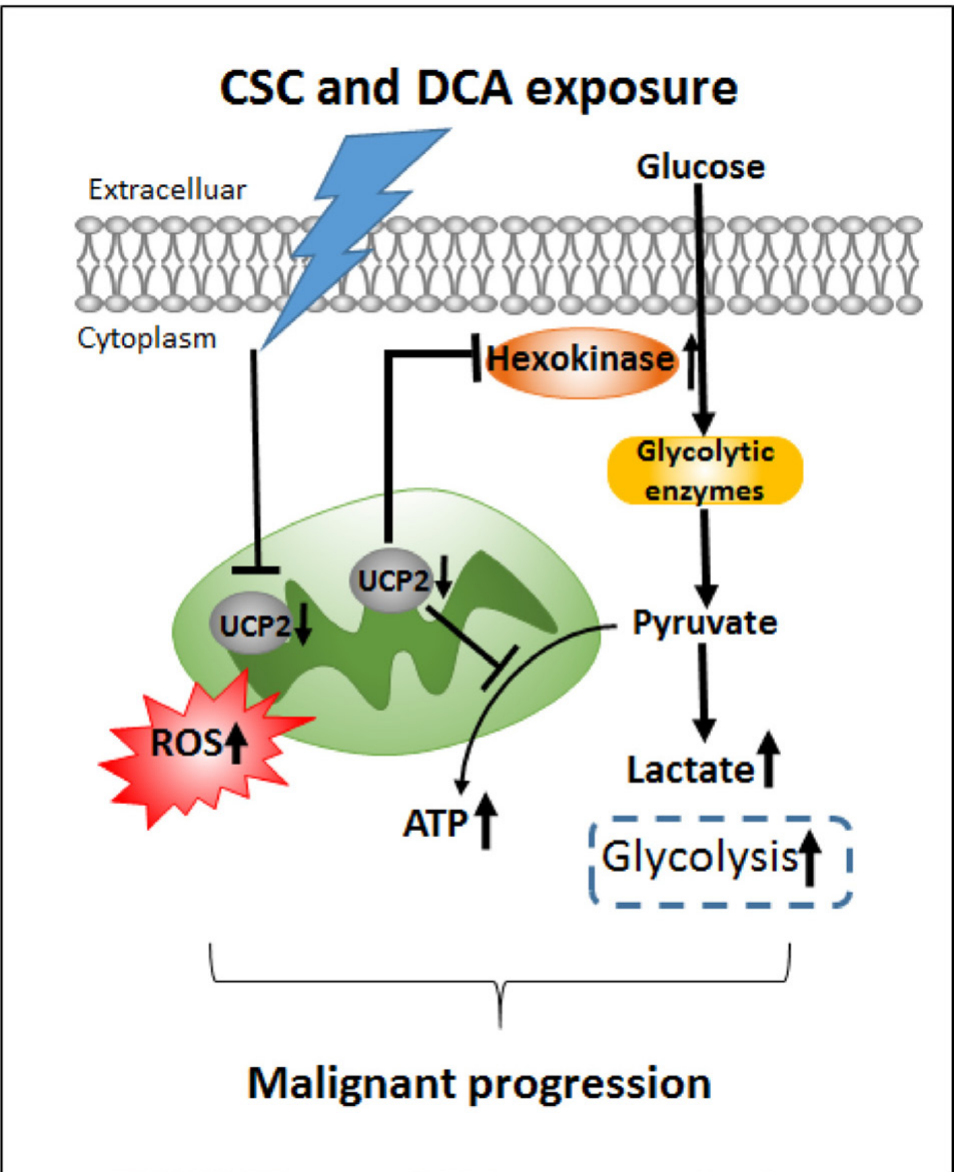
A schematic illustration of a role of UCP2 in bile acids and cigarette smokes-induced metabolic regulation Downregulation of UCP2 due to CSC and DCA exposure increases ROS generation and promotes glycolysis though increasing Hexokinase 2, leading to EAC malignancy progression.

Our results provide the first evidence that metabolic changes in EACC are associated with the aggressive phenotype following exposure to DCA and CSC. DCA and CSC appear to alter the metabolism of EAC cells via degradation of UCP2 and re-expression of UCP2 abrogated the effects of DCA and CSC (Figure 7). While targeting UCP2 directly for EAC therapy would be difficult, these findings do suggest that shifting the mitochondrial changes back toward a normal state is a potential novel strategy. Collectively, our findings support further studies of mitochondrial cellular energetics in EAC and the development of metabolic strategies for the treatment of these neoplasms. Further work is our laboratory is ongoing in this area.

## MATERIALS AND METHODS

### Cell culture

NCI-SB-Esc2 (Esc2) is an EAC cell line established at the National Cancer Institute, NIH that has been reported in the literature (Laboratory of Dr. David S. Schrump) [44]. OE33 (JROECL33) and FLO-1 are commercially available EAC cell lines purchased from European Collection of Authenticated Cell Cultures (ECACC, UK). OE33 and FLO-1 were obtained in 2014. All cell lines were authenticated with human leukocyte antigen (HLA) analysis by NIH-HLA laboratory (Supplementary Table 1). All cell lines were tested by PCR for mycoplasma contamination every six months. All cells were cultured in RPMI media supplemented with 10% fetal calf serum (FCS) and 1% penicillin/streptomycin (normal media).

### Treatment conditions

Deoxycholic acid (DCA) was purchased (Sigma) and suspended at a concentration of 500 mM in DMSO as stock concentration. Cigarette smoke condensate (CSC) was derived from Kentucky Reference 3R4F research blend cigarettes (University of Kentucky, Tobacco and Health Research Institute, Lexington, KY, USA), using a Borgwaldt-LM1 smoking machine (Richmond, VA, USA) and standard Federal Trade Commission smoking conditions (35 ml puff volume, 2.0 s duration, and 1 puff/min, 9 puffs/cigarette). The smoke condensates were trapped on Cambridge glass fiber filters, weighed, and re-suspended at a concentration of 50 mg/ml in DMSO as stock concentration. For DCA and CSC exposure experiments, cells were cultured in 10-cm dishes in appropriate normal media (NM) with DMSO, or NM with CSC (10 μg/ml), or NM with DCA (100 μM), or NM with combination. These conditions were established based on no reduction in proliferation or viability. Medium was changed daily with the addition of fresh CSC, DCA, or DMSO control. Cells were harvested at various time-points for further analysis.

### Anchorage-independent growth

Cells were trypsinized to generate a single-cell suspension and seeded in 6-well plates with under-layers of 0.70% agarose in RPMI medium supplemented with 10% FCS. Soft-agar plates were prepared to test capacity for colony growth. Cells were plated in triplicate at a density of 1×10^4^ in 1ml of 0.35% agarose over the agar base. Cultures were fed every 7 days. After 3 weeks, media was removed and cells rinsed carefully with PBS. One ml 1% crystal violet added for staining for 1h. Colonies with >50 cells were counted.

### Invasion assay

Invasion of cells was evaluated using Transwell^TM^ chambers with 8 μm pore filters (Corning Inc., Corning, NY) in 24-well plates. To assess the capacity for invasion, 5 × 10^4^/100 μl cells were added to upper chambers that had been coated with 35 μl of Matrigel (BD Biosciences, Franklin Lakes, NJ) in supplement-free medium. Normal RPMI medium was added to the lower chamber as a chemoattractant. The chambers were incubated at 37°C with 5% CO_2_ for indicated time points. At the end of incubation, cells on the upper surfaces of the filters were removed with a cotton swab. Cells which invaded through the filter to the lower surface were stained with crystal violet for 10 min. Invading cells were viewed and photographed under a phase contrast microscope (Olympus, Tokyo, Japan). 100 μl extraction solution was added to transfer to 96-well plate for measurements in the plate reader at OD 560 nm.

### Wound-healing assay

Cells were seeded in 6-well plates and maintained at 37°C and 5% CO_2_ for 24 h to permit cell adhesion and the formation of a confluent monolayer. Wounds were made by passing a plastic tip across the monolayer cells. The cell surface was then washed with serum-free culture medium three times to remove dislodged cells. Wound closure was monitored by collecting digitized images at indicated time points after the scratch was performed. The time of wound infliction was considered as 0 h and wound closure was monitored for up to 24 h. Wound closures were photographed by phase contrast microscopy (40X) at indicated time points after scraping. Covered areas by migrating cells (%) were determined by Image J software and the following equation: ((Wound area at 0 h - Wound area remaining after 24 h)/Wound area at 0 h x100%).

### Quantitative RT-PCR

Total RNA was prepared using TRIzol reagent (Invitrogen). 1 μg of total RNA was reverse transcribed using iScript reverse transcriptase (Bio-Rad; Hercules, MD). Omission of reverse transcriptase served as a negative control. cDNA was amplified using Platinum PCR SuperMix (Invitrogen). Real-time quantitative RT-PCR analysis was done using hexokinase 2 (HK2), UCP2, and β-actin primers from Applied Biosystems or Integrated DNA Technologies. β-actin was used as an internal control. Fold changes in expression of each gene were calculated by a comparative threshold cycle (Ct) method using the formula 2^–(ΔΔCt)^.

### Western blots

Total cell lysates were prepared with a detergent buffer. Protein concentrations were measured with the BCA Protein Assay per the manufacturer's manual (Thermo Fisher Scientific). Equal amounts of protein were separated by 10% sodium dodecyl sulfate-polyacrylamide gel electrophoresis and were transferred to polyvinylidene fluoride (PVDF) membranes (Millipore, Billerica, MA). Membranes were incubated overnight at 4°C with a 1:1000 dilution of antibodies for β-Actin (Santa Cruz, CA), UCP2 and HK2 (Abcam). After additional incubation with a 1:1000 dilution of an anti-immunoglobin horseradish peroxidase-linked Ab for 1 h, the immune complexes were detected by the SuperSignal^®^ West Femto Maximum Sensitivity Substrate (Thermo Fisher Scientific). Western blot quantification and normalization to actin following user guide by Image Lab.

### Cellular ROS detection assay

Cellular ROS detection assay (Abcam PLC, Cambridge, MA, USA) was used to measure hydroxyl, peroxyl, and other ROS activity within the cell following the manufacturer's instructions. Cells were seeded into each well and cultured for at least 4 h before measurements. Cells were washed once with PBS supplemented with 2′, 7′-dichlorofluorescein diacetate (DCFDA) or Deep Red Fluorescence. After incubation at 37°C for 1h, cells were resuspended in PBS in the presence of freshly prepared 10% FBS followed by another 30 minute culture at 37°C. The cells were analyzed on FACS Calibur flow cytometer (BD Biosciences). Relative fluorescence intensities were analyzed and histograms generated using Cell Quest.

### ATP assay

The level of intracellular ATP was measured by using a ATP bioluminescence assay kit (Roche Life Science, IN). ATP levels were determined following the manufacturer's instructions. Briefly, 1 × 10^6^ cells were lysed in the 100 μl TE buffer and boiled for 5 min at 100°C. Samples centrifuged at 3000 × g for 5 min to pellet insoluble materials. 50 μl supernatant was added to a 96-well, black-sided, optical clear-bottom plates (Corning, NY), and followed by the addition of 50 μl of the luciferin/luciferase reagent to each well. Luminescence was measured in Synergy microplate reader (BioTek, VT). Bioluminescence actives were normalized to the cell numbers.

### Lactate assay

Lactate levels in the supernatants of cell cultures were measured with Lactate Colorimetric / fluorometric assay kit (BioVision, CA) following the manufacturer's instructions. Briefly, cell medium was collected and frozen in –80°C overnight, the standard curve was made with lactate at 0, 20, 40, 60, 80, and 100 μM. The correlation coefficient was 0.995 or higher. 1 μl cell media was added to a 96-well plates and followed by the addition of 49 μl of assay buffer. 50 μl lactate reaction mixture was added with the enzyme mix and the probe reagent to each well. After 30 min of incubation at room temperature, the reading of absorbance (OD 570 nm) was taken using Synergy microplate reader. The relative lactate concentration was normalized by the cell numbers or sample's protein concentration.

### Lentiviral transfection

UCP2 (TRCN0000060144) or scramble shRNAs in the pLKO lentiviral vector were purchased (Sigma). The cDNA for UCP2 cloned into a lentiviral vector (pLenti-GIII-CMV-GFP-2A-Puro) with CMV promoter were obtained from Applied Biological Materials (BC, Canada). Cells were sub-cultured at 1.0 × 10^4^ cells/well into 96-well plate. Incubate 18–20 hours at 37°C in a humidified incubator in an atmosphere of 5% CO_2_. The viral supernatant was then added into cells at a multiplicity of infection (MOI) of 10. After 48–72 h of incubation, the cell culture medium was changed with 2 μg/ml puromycin. Images of the cells expressing GFP were captured under a phase contrast microscope.

### Murine xenograft experiments

Esc2 cells were transfected with pLenti-GIII-CMV-GFP-2A-Puro vector control. pLenti-UCP2 were suspended in phosphate-buffered saline (PBS) at a concentration of 5 × 10^5^ cells/100 μL for flank injections. UCP2-transfected cells were injected subcutaneously into the right flank of 7-week-old female, athymic, nude mice, whereas the other flank was injected with the same number of control transfected cells (10 mice / 20 flanks per experiment). Mice were monitored once every day and tumor size was measured every other day. Tumor volumes were calculated by the formula V = ½ × L × W^2^ (L - length (longest dimension); W - width (shortest dimension)) [45]. Approximately 15 to 30 day later mice were euthanized, evaluated for percent tumor take, and mass of excised xenografts. Tumor tissues were snap frozen in liquid nitrogen. All animal procedures were approved by the National Cancer Institute Animal Care and Use Committee and were in accordance with the NIH Guide for the Care and Use of Laboratory Animals.

### Statistical analysis

Quantitative data were presented as the mean ± SD. The Student *t*-test was carried out to compare means between two groups. One-way ANOVA followed by the post hoc Dunnett test was used to compare means of more than two group, and a multiple range least significant difference (LSD) was used for intergroup comparisons. Results with *P* < 0.05 were considered statistically significant and are indicated by ^*^, ^#^, ^Δ^, *P* < 0.05; and ^**^, ^##^, ^ΔΔ^, *P* < 0.01. All statistical analyses were performed with SPSS 16.0.

## SUPPLEMENTARY MATERIALS FIGURES AND TABLES


