# Supplementary material for: Bile acid and cigarette smoke enhance the aggressive phenotype of esophageal adenocarcinoma cells by downregulation of the mitochondrial uncoupling protein-2
Source: Oncotarget. 2017 Nov 10;8(60):101057–71. doi: 10.18632/oncotarget.22380 (PMC5731855; doi:10.18632/oncotarget.22380)
Supplement: Supplementary file 1 [file oncotarget-08-101057-s001.pdf]

Bile acid and cigarette smoke enhance the aggressive phenotype of esophageal adenocarcinoma cells by downregulation of the mitochondrial uncoupling protein-2

SUPPLEMENTARY MATERIALS

Cell proliferation assays

The cell proliferation rate was measured using a CyQUANT proliferation assay (Life Technologies #C7026) according to the manufacturer's instructions. After different treatments, cells were plated at equal concentrations in 96-well plates. For the following 5 days, each day media was carefully removed from wells without disturbing the cells and each plate was stored at –80°C. Exactly 200 ul of fluorescent CyQUANT GR dye, which exhibits strong fluorescence enhancement when bound to cellular nucleic acid, was added to each well. Plates were incubated at room temperature for 10–20 min. Cellular DNA was quantified using a fluorescence spectrophotometer (BioTek, VT) with excitation at 480 nm and emission detection at 520 nm.

JC-1 assays

Mitochondrial membrane potential ($\Delta\psi m$) changes were detected using the mitochondrial membrane potential-sensitive fluorescent dye JC-1 (Thermo Fisher Scientific) following the manufacturer's instructions. After different treatments, cells were collected and suspended in PBS at approximately 1×10^6 cells/ml containing JC-1 and the cells incubated at 37°C, 5% CO_2 for 30 min. After loading, the cells were washed twice with PBS and analyzed by flow cytometry (FACS Caliber, Becton Dickinson, Heidelberg, Germany). The JC-1 monomer (green) and the J-aggregates (red) were detected separately in FL1 (emission, 525 nm) and FL2 (emission, 590 nm) channels, respectively. The red/green emission ratio provided an estimate of the $\Delta\Psi m$.

Supplementary Table 1: HLA typing for cell lines

Name	Details	HLA typing
**NCI-SB-Esc2**	Tumor from a paraaortic lymph node in a 54-year-old male with a history of tobacco and alcohol use.	A02, 68, B14, 44, C05, 08
**FLO-1**	Primary distal esophageal adenocarcinoma in 68-year-old caucasian male.	A02, B40, 44, C02, 05
**OE33**	(JROECL33). 73 y/o female with stage IIA, lower esophageal adenocarcinoma arising in Barrett's metaplasia.	A01, B08, C07

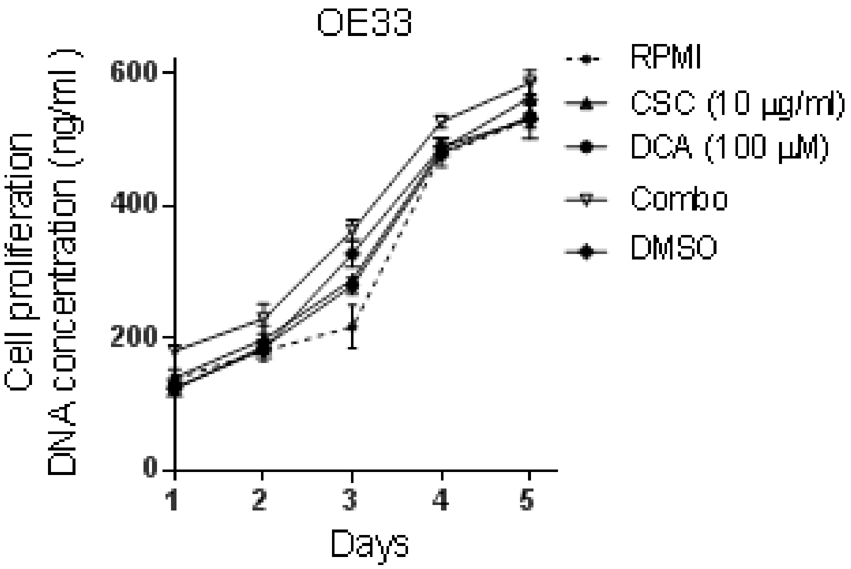

Supplementary Figure 1: Bile acids and cigarette smoke have no effects on esophageal cancer cell proliferation. OE33 cells cultured in the presence or absence of CSC and/or DCA for 5 days. OE33 cell proliferation was measured by CyQuant assay in which DNA concentration is a measured as a surrogate for cell numbers. Combo stands for the group with DCA and CSC combined treatment.

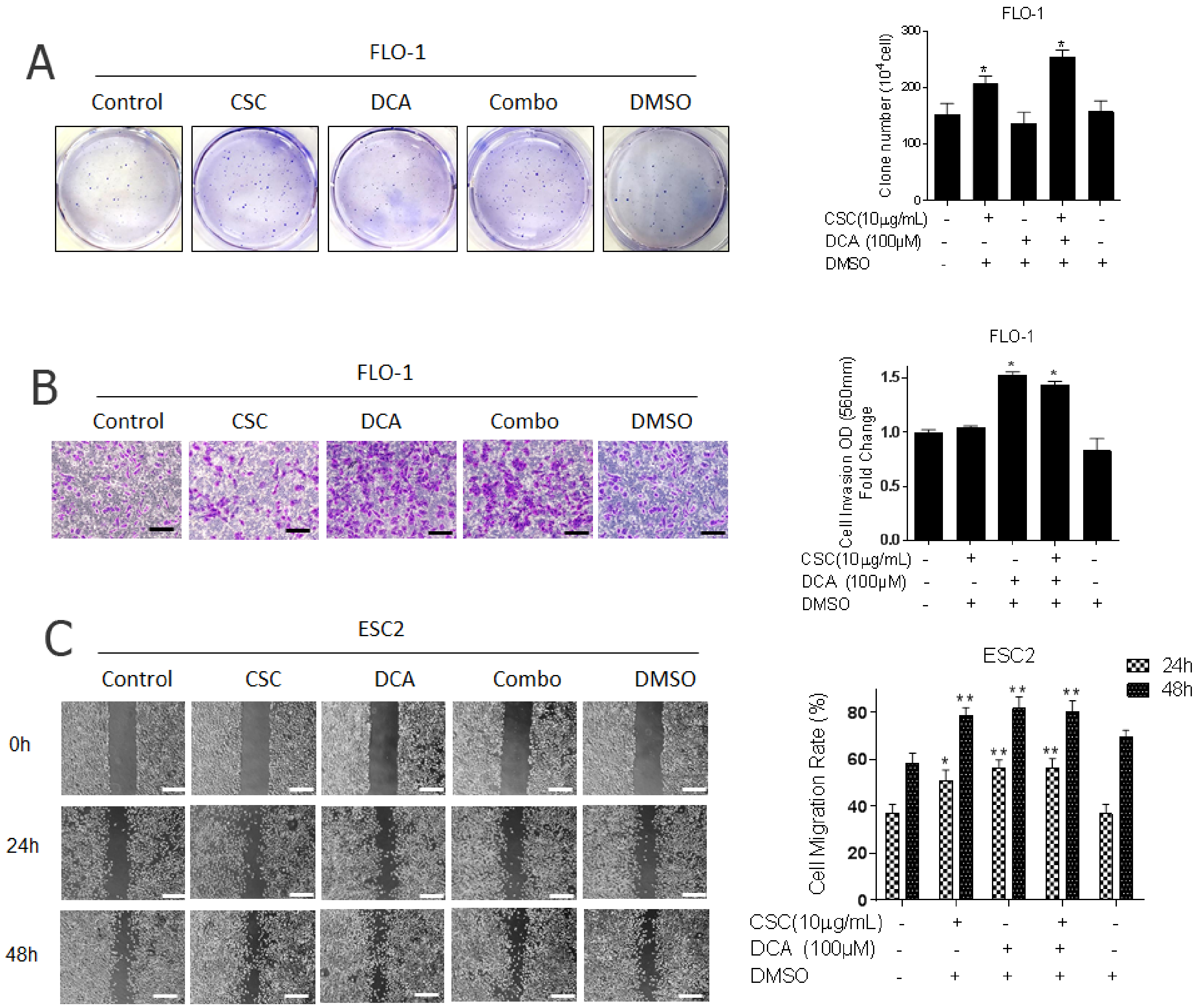

Supplementary Figure 2: Bile acid and cigarette smoke enhance the aggressive phenotype of esophageal adenocarcinoma cells. FLO-1 and Esc2 cells cultured in the presence or absence of CSC and/or DCA for 5 days. (**A**) The colonies in soft agar were stained with crystal violet, photographed, and counted to quantify (mean ± SD) in FLO-1 cells. Three independent experiments were performed. (**B**) Invasion was determined by transwell assays in FLO-1 cells. Representative image of invading cells is shown (original magnification × 100, scale bar = 50 μm). Quantitative analysis of invasion was measured by absorbance at OD 560 nm after staining of invading cells with crystal violet. Fold changes (mean ± SD) were obtained from three independent experiments. (**C**) The cell migration was analyzed wound-healing assays in Esc2 cells. Photographs were obtained at 0 h (immediately after scratching) and at the indicated time intervals shown (original magnification × 40, scale bar = 125 μm). Covered areas by migrated cells in the nine random fields after exposure for 0, 24, 36 h were quantified by Image J software. $^{*}P < 0.05$ and $^{**}P < 0.01$ as compare with non-treatment group.

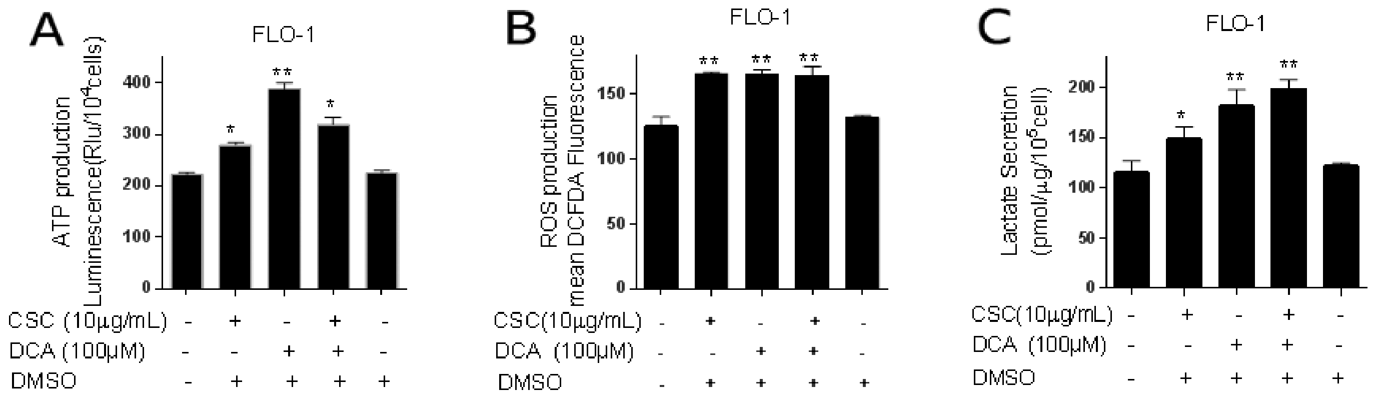

Supplementary Figure 3: Bile acid and cigarette smoke alter mitochondrial function and promote glycolysis. FLO-1 cells were cultured in the presence or absence of CSC and/or DCA for 5 days. (**A**) ATP levels were measured by luciferase luminescence intensity which was normalized to the cell number (mean ± SD) from three independent experiments. (**B**) The level of cellular ROS concentrations was measured using DCFDA fluorescence by flow cytometry with the geometric mean of fluorescence ± SD analyzed using Cell Quest software. (**C**) Lactate released into the culture medium were measured by absorbance at OD 560 nm and normalized to the cell numbers (mean ± SD) from three independent experiments. $^{*}P < 0.05$, and $^{**}P < 0.01$ as compare with control culture group.

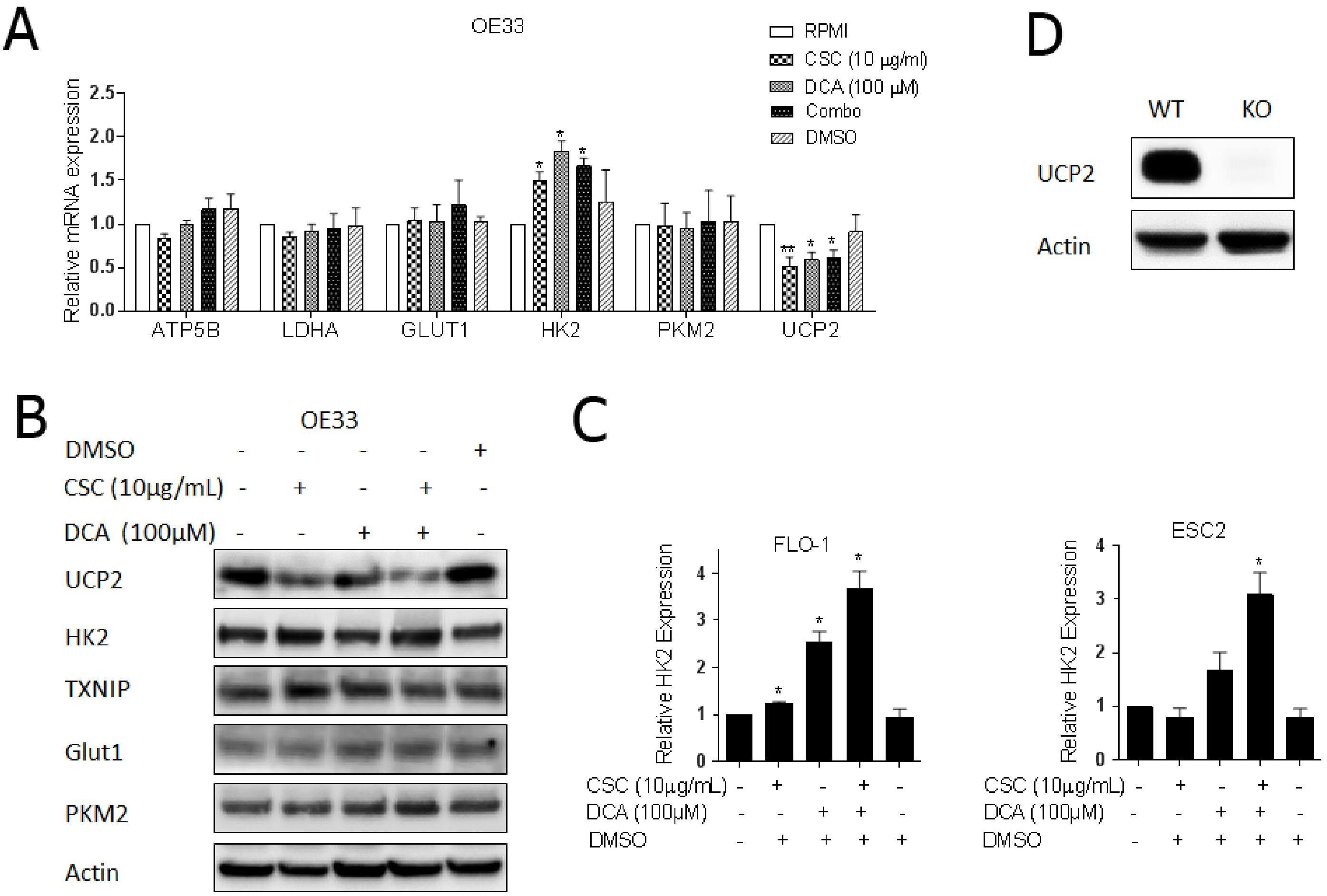

Supplementary Figure 4: Bile acids and cigarette smoke effects on metabolically-associated gene expression in esophageal cancer cells. OE33 cells were cultured in the presence or absence of CSC and/or DCA for 5 days. (**A**) qRT-PCR analysis of metabolically-associated gene expression normalized with actin in OE33 cells. Combo stands for the group with DCA and CSC combined treatment. (**B**) The protein levels of metabolically-associated gene expression were analyzed by Western blots. (**C**) qRT-PCR analysis of HK2 normalized with actin in FLO-1 and Esc2 cells. (**D**) The protein levels of UCP2 were analyzed by western blots with UCP2 antibodies in WT and UCP2 KO mice spleen. $^{*}P < 0.05$, and $^{**}P < 0.01$ as compare with control culture group.

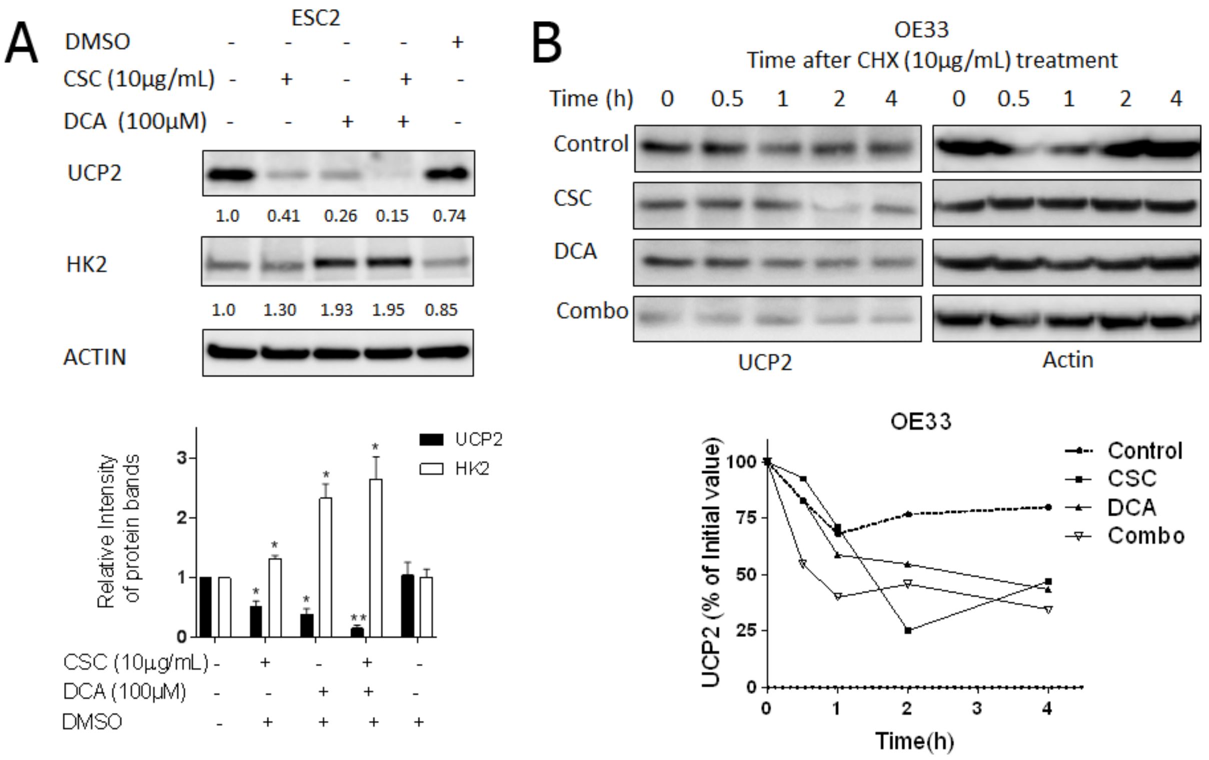

Supplementary Figure 5: Bile acids and cigarette smokes promotes UCP2 protein degradation as result of UCP2 downregulation. Esc2 cells were cultured in the presence or absence of CSC and/or DCA for 5 days. (**A**) The protein levels of UCP2 and HK2 were analyzed by Western blots in Esc2 cells. The relative protein levels of UCP2 were quantified by Image lab software and corrected for loading control β-actin. Quantification of UCP2 expression is expressed relative to control group. $^{*}P < 0.05$, and $^{**}P < 0.01$ as compare with control culture group. (**B**) After OE33 cells were exposed to CSC and DCA for 5days, the cells were treated with protein synthesis inhibitor cycloheximide (CHX, 10 μg/ml) for the times indicated. Levels of UCP2 were determined by western blots. The relative protein levels of UCP2 were quantified by Image lab software and corrected for loading control β-actin. The densitometry quantification of UCP2 compared with the initial quantification are presented at different time points. Combo stands for the group with DCA and CSC combined treatment.

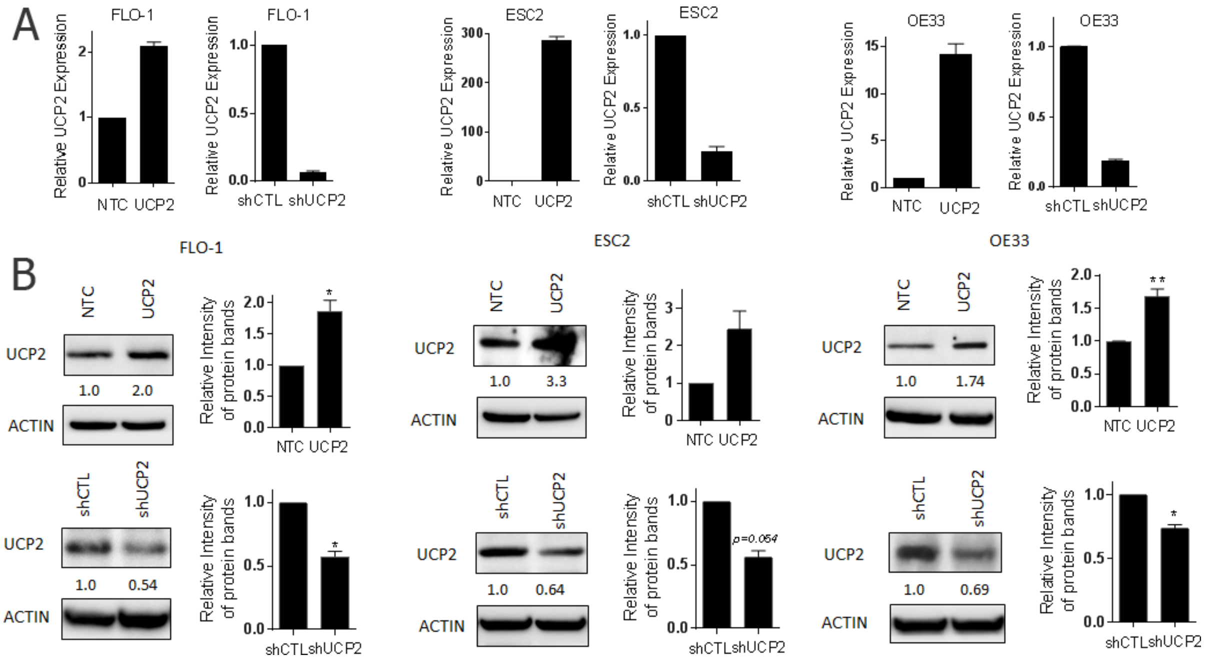

Supplementary Figure 6: UCP2 overexpression and knockdown via lentiviral transfections. EAC cells were transfected to stably expressing non-targeting control (NTC) or UCP2 lentiviruses. Stable knockdown of UCP2 (shUCP2) or scrambled control (shCTL) were generated by transfecting short-hairpin RNA lentiviruses. (**A**) qRT-PCR analysis of UCP2 expression normalized with actin in FLO-1, Esc2, and OE33 cells with or without UCP2 transfection. (**B**) Western analysis of UCP2 expression in UCP2 overexpressed and knockdown cells. The relative protein levels of UCP2 were quantified by Image lab software and corrected for loading control β-actin. Quantification of UCP2 expression is expressed relative to control group. $^{*}P < 0.05$, and $^{**}P < 0.01$ as compare with control culture group.

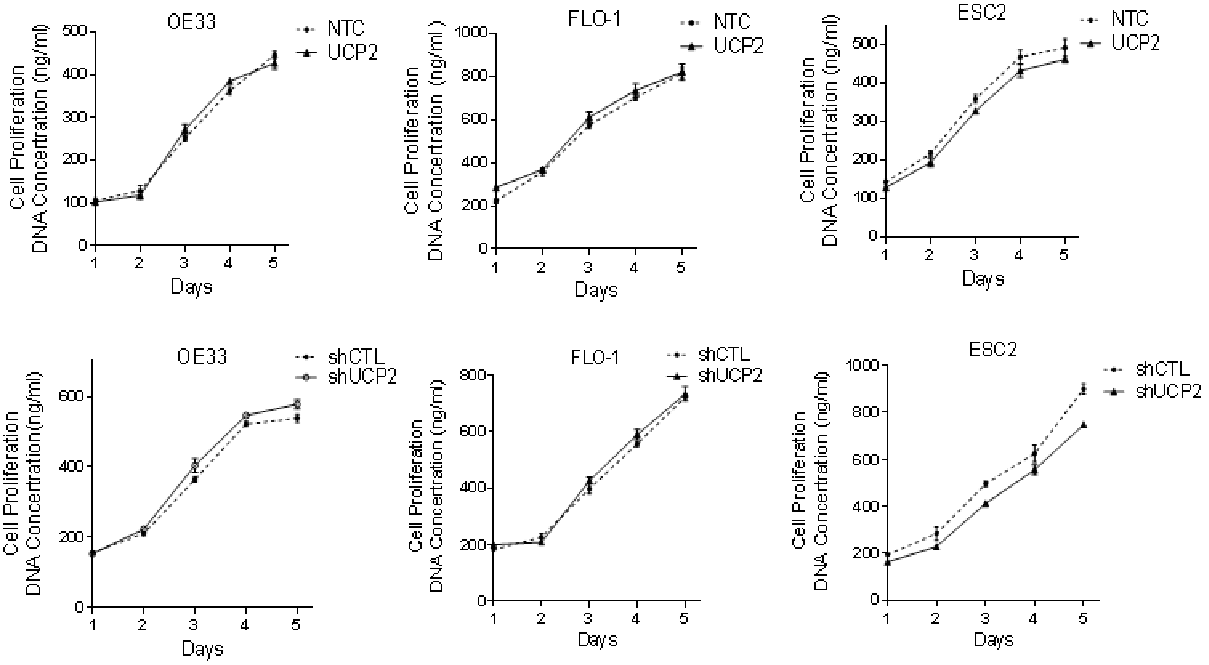

Supplementary Figure 7: UCP2 had no effects on the esophageal cancer proliferation. EAC cells were transfected to stably expressing non-targeting control (NTC) or UCP2 by UCP2 lentiviruses. Stable knockdown of UCP2 (shUCP2) or scrambled control (shCTL) were generated by transfecting short-hairpin RNA lentiviruses. EAC cell proliferation was measured by CyQuant assay in which DNA concentration is a surrogate for cell numbers.

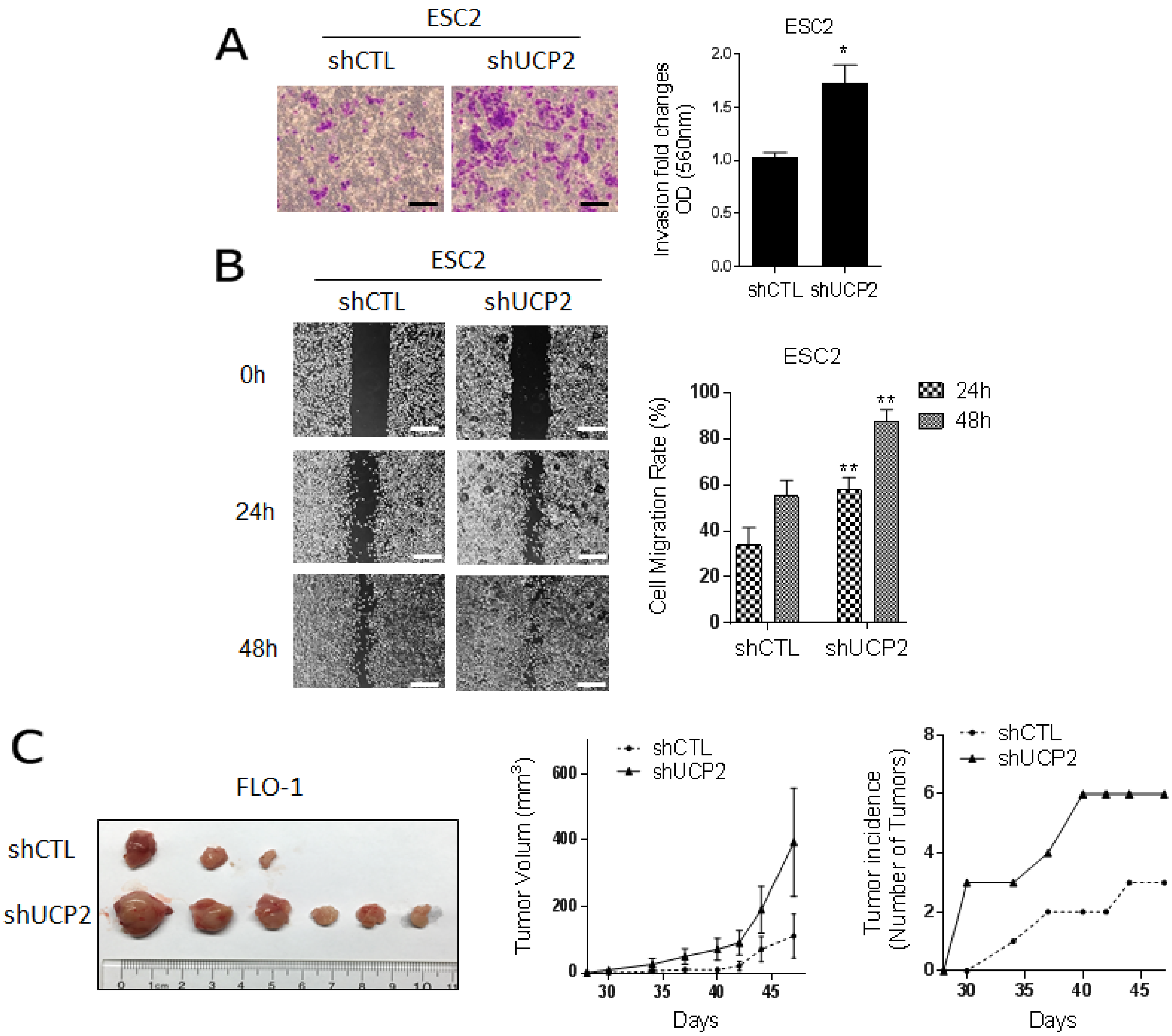

Supplementary Figure 8: UCP2 impairs malignant progression of esophageal cancer cells. FLO-1 and Esc2 cells were generated with stable knock-down of UCP2 (shUCP2) or scrambled control (shCTL) by transfecting short-hairpin RNA lentiviruses. (**A**) Invasion was determined by transwell assays. Representative image of invading cells is shown (original magnification × 100, scale bar = 50 µm). Quantitative analysis of invasion was measured by absorbance at OD 560 nm after staining of invading cells with crystal violet. Fold changes (mean ± SD) were obtained from three independent experiments. (**B**) The cell migration was analyzed with wound-healing assays. Photographs were obtained at 0 h (immediately after scratching) and at the indicated time intervals shown (original magnification × 40, scale bar = 125 µm). Covered areas by migrated cells in the nine random fields after exposure for 0, 24, 48h were quantified by Image J software. $^{*}P < 0.05$, and $^{**}P < 0.01$ as compare with control group. (**C**) Xenograft experiments were performed and Esc2 cells with UCP2 or control transfection, cells were injected subcutaneously into flanks of nude mice (10 mice / 20 flanks per experiment). Photographs of representatives harvested tumors derived from each group. Tumor growth curve in nude mice measured by caliper (mean ± SD), and tumor incidence was calculated.

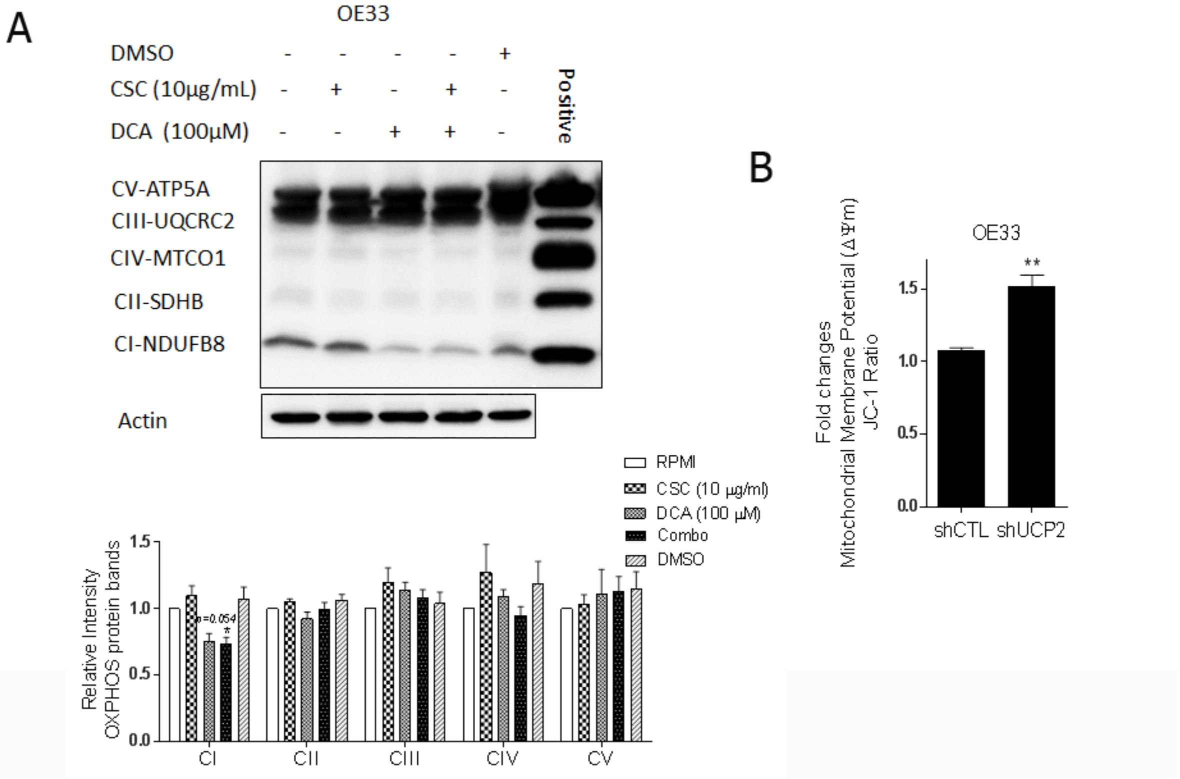

Supplementary Figure 9: Bile acid and cigarette smoke condensate have no effects on the mitochondrial respiratory chain but uncoupling activity of UCP2 OE33 cells cultured in the presence or absence of CSC and/or DCA for 5 days. (**A**) The protein levels of mitochondrial complexes were analyzed by Western blots with total OXPHOS antibody cocktail staining, rat heart mitochondrial sample as a positive control. The relative protein levels of CI, CII, CIV, CIII and CV were quantified by Image lab software and corrected for loading control β-actin. Quantification of CI, CII, CIV, CIII and CV expression is expressed relative to control group. $^{*}P < 0.05$ as compare with control culture group. OE33 cells were generated with stable knock-down of UCP2 (shUCP2) or scrambled control (shCTL) by transfecting short-hairpin RNA lentiviruses. (**B**) Analysis of mitochondrial membrane potentials using flow cytometry with JC-1 dye. Loss of $\Delta\Psi$m was demonstrated by the change in JC-1 fluorescence from red (JC-1 aggregates) to green (JC-1 monomers). The data (mean ± SD) showed the ratio of JC-1 red fluorescence to green fluorescence. $^{**}P < 0.01$ as compare with control group.
